# The Institutional Worker

**Published:** 1920-02-07

**Authors:** 


### INSTITUTIONAL WORKER

Being a Special Supplement to "The Hospital"

### OUR BUREAU OF INFORMATION.

### Rules for Correspondents.

1. Every letter must be accompanied by the coupon to be cut from the back cover (inside page) of The Hospital, current issue, and must contain the name and address of the correspondent with pseudonym for publication if desired. Replies by post cannot be given save under exceptional circumstances at the Editor's discretion.
2. Letters from Approved Homes in reply to special needs published in the Bureau should state terms and full particulars, and be sent prepaid under cover to the Editor of the Bureau with name written across coupon for identification.
3. Proprietors of Homes which have not yet been entered on the List of Approved Homes, but have spare accommodation likely to suit special needs, are invited to write for an application form for registration. The fee for registration, which includes two announcements of the Home in the Bureau and other privileges, is 10s.
4. All communications to be addressed to the Editor of The Hospital, 28 Southampton Street, Strand, London, W.C. 2, and marked "Bureau of Information."

### INSTITUTION FACTS AND FIGURES.

### WELFARE WORK ADMINISTRATION.

Points on administration of welfare work are contained in a report of the Hull medical officer of health, who explains the method in Hull.

The midwives inspector exercises general supervision over the work of the midwives practising in the city, carries on general supervision of the work with regard to the Notification of Births Act, and supervises the work of the health visitors.

For the purpose of arranging the duties of the health visitor, the city is divided into nine districts. On receipt of a birth notification a visitor proceeds to the house and obtains particulars. Advice is given as to the general care of the infant and mother and all other children in the house up to five years of age. As far as possible revisits are made to the home to ascertain the progress of the infant and the other children, and where it is found there is a deviation from normal health, medical attention is advised at one of the clinics or elsewhere, as the case may require.

Expectant mothers are advised and encouraged to attend the nearest clinic, unless their health is such as to prohibit this.

Investigation is also made into all cases of ophthalmia neonatorum. These cases require frequent revisits to see that the infant is kept under medical care until the termination of the disability. Cases of puerperal fever, whooping cough, diarrhœa, and cerebro-spinal meningitis are investigated, and the usual printed and verbal instructions given as to precautions to be adopted.

Reports of neglected children and aged people are inquired into, and when advisable the Poor-law officers, school attendance officers, or the S.P.C.C. are notified.

The health visitors are also required to make a note of nuisances discovered or complained of at the homes they visit, and to forward a report to the Chief Inspector of Nuisances.

The time spent at clinics—of which there are now two—by the health visitors varies with the attendance of mothers and children, but the average is about three hours per day for each visitor on three days a week. Before the arrival of the medical officer one health visitor interviews the mother and enters the name of the child or mother, or expectant mother, or both, as the case requires. The case is then passed on to the second health visitor, who weighs the child and records its weight, and then refers it to the medical officer. A third health visitor tests the urine of expectant mothers and attends upon the medical officer. If the latter advises Virol or malt extract, a recommendation slip is made out and taken to the first health visitor, who sees that the medical officer's instructions are carried out. The name, address, and other particulars are noted in a special book, and the mother advised when to revisit. When there are only two health visitors at a clinic these duties must be undertaken by them. Prepared milk is sold only to those mothers for whose infants it is prescribed, and the amount of milk is in accordance with the medical officer's instructions.

A free supply of milk is given in necessitous cases on the recommendation of the medical officer after an investigation has been made. Much time is taken up in investigating applications for free midwives and for admission to the maternity home.

The health visitors report daily to the inspector of midwives and receive further instructions as to their work. On Saturdays they submit a written report on their week's work to the superintendent, and make certain preparations for the following week's work.

Ante-natal visiting is very essential, and great importance is attached by the Ministry of Health to the systematic revisiting of children at their homes, but with the present staff of health visitors it is not possible to keep up to the standard desired. The Ministry of Health, in the light of experience, considers that the standard of 500 births to each health visitor which had been previously suggested should be modified, and suggests that a district with about 400 births a year will be as much as one visitor can undertake unless the district is very compact or of such a class that many infants do not need visiting.

### Saving Fuel in Boiler House.

The importance of protecting all boilers, steam and hot-water pipes with suitable non-conductive material is more than ever emphasised by the present-day price of fuel. Although this is a matter which, as a general rule, receives attention in hospitals and other institutions, it is nevertheless a fact that defective boiler lagging and unprotected pipes not infrequently escape notice. It is said by experts on the subject that the condensation in an installation of steam boilers, cylinders, and pipes which are unprotected is equivalent to nearly 1 lb. of water per square foot per hour, and the loss of heat resulting from this condensation corresponds under normal conditions to about 2 oz. of coal per square foot per hour. Thus it may be shown that a small area uncovered may be responsible for the wastage of many tons of coal in the course of a year. We feel sure that if these facts were realised by those whose heating installations are not controlled by skilled engineers a great saving of fuel would be effected.—K. H.

### Question for February.

### A NURSE'S VENTURE AS OPTICIAN.

**A trained nurse with long experience in an eye hospital, who has taken the certificate of the British Optical Association, is desirous of setting up business in a flourishing provincial town. Give suggestions and advice as to size of shop, its fittings, capital required, staff required, and the best method of making the shop known by advertising or otherwise.**

**(A minimum payment of 10s. 6d. will be made for each published answer.)**

### RULES.

The following rules must be observed :—

1. Contributions must be written on one side of the paper. Conciseness and terseness are desirable features. The MS. must bear the name and address of the sender and be accompanied by coupon to be cut from the back cover (inside page) of the current issue of THE HOSPITAL. A pseudonym must be chosen if the name is not to be published.

2. Contributions must be addressed to the Editor of THE HOSPITAL Institutional Worker Supplement, 28 & 29 Southampton Street, Strand, London, W.C. 2, should reach him before the end of the current month, and be marked in the left-hand corner "Facts and Figures."

### QUESTIONS INVITED.

IN connection with our Question Box, we offer every month five shillings each for the best questions which are sent in for consideration. The questions may be on any institutional subject and concern any department, from the secretary's to the porter's, or the matron's to the domestic staff; the one essential is that they must be practical and deal with points that have a definite relation to hospital or institutional administration, upkeep, finance, or management. Points relating to artificers' work, housekeeping, the laundry, out-patients, and other practical matters will be welcome. It is hoped that thus, when difficult or doubtful points arise in the course of their work, institutional workers will be encouraged to put them in the form of a question and send them to the Editor, THE HOSPITAL Bureau of Information, 28 & 29 Southampton Street, Strand, London, W.C. 2, marked "Question Box."

The best questions will be published in due course, and our readers will be given the opportunity of answering them. Thus all institutional workers may be able to cooperate with the view of helping the work and smoothing the difficulties of each other. In short, we want the Question Box of the *Institutional Worker* to be freely used by every worker habitually.

### ENQUIRIES AND ANSWERS.

### *THE SICK AND IN NEED.*

### Home for Orphan Girl.

YOU might write to the secretaries of the following homes on behalf of the small girl whom you wish to place in a home where she will be trained in all kinds of housework: The Mount Hermon Girls' Orphan Home, Park Lane, Sevenoaks, Kent. Children are admitted from five to sixteen years of age. At the age of sixteen they are placed in suitable situations. Royal Female Orphan Asylum, Beddington, near Croydon, Surrey (the office address is 17 Buckingham Street, Strand, W.C.). Age of admission is from seven to ten; inmates are retained until sixteen years of age; and the Children's Home, Nether Street, Finchley, N. Give full particulars of the case when making your application. —MRS. C.

### Emergency Nurses.

YES; the scheme for providing emergency nurses in London has now matured, and all applications for such help should be made to the Assistant Secretary of Central Council for District Nursing in London, Miss Pollock, c/o the City Parochial Foundation, 3 Temple Gardens, London, E.C. 4.—SUPERINTENDENT.

### *EMPLOYMENT AND TRAINING.*

### Nursing Homes in Edinburgh.

THE following are the addresses you ask for: Royal Scottish Nursing Institution, 19 Drumsheugh Gardens, Edinburgh; and the Co-operative Association, 2 Melville Street, Edinburgh.—SCOTTIE.

### Nursing in New Zealand.

WE suggest that you write to the Agent-General for New Zealand, 13 Victoria Street, London, S.W. 1, who might be able to help you. You might also write to the Superintendent of the Trained Nurses' Association, Wellington, New Zealand.—C. W. S.

### Stokehold Drainage.

THE trouble you experience in your stokehold through the drainage of surface water into it is not an uncommon one, but unless it be effectively dealt with damage may be done to the beds of your boilers, and even to the boiler plates when the boilers are not in use. Your trouble seems to be that the level of the water in your sump is not low enough to keep your surface drain clear, and if you have no drain low enough in which properly to drain the sump the best course to adopt would be to instal a small motor-driven pump capable of raising the water in the sump to some drain at a higher level. A small centrifugal pump driven by an electric motor would probably be the best type to use. The Worthington Pump Co., Ltd., 153 Queen Victoria Street, London, E.C., make an excellent pump of this kind.—KOROA.

### Training in Ophthalmic Hospital.

YES, the training at a good ophthalmic hospital would be quite useful to you until you are old enough to enter a large hospital for general training. You might apply to the Royal London Ophthalmic Hospital, City Road, E.C. 1, or to the Royal Eye Hospital, St. George's Road, Southwark, S.E.—EAGER.

### Work Among the Blind, Deaf and Dumb.

YOUR best course would be to apply to the National Institute for the Blind, 224-228 Great Portland Street, W. 1, who always give advice to those interested in the well-being of the blind, who desire to become workers in this sphere of usefulness. The National Association for the Oral Instruction of the Deaf, 11 Fitzroy Square, W., undertake the instruction of those who wish to become teachers, and they have established a training college for this purpose.—E. M. P.

### Electro-Medical Training.

ONLY those who have passed the examination of the Incorporated Society for Trained Masseuses, qualifying them as masseuses, can enter for the electro-medical examination, which is held every three months by the same Society. The training extends over a period of three months, during which time a stated number of hours must have been spent in gaining practical knowledge in a hospital. Write to the Secretary of the Incorporated Society of Trained Masseuses, 157 Great Portland Street, London, for full particulars.—T. N. A

### *MISCELLANEOUS.*

### Medals for War Service.

YOUR war service at home does not count for the Victory Medal; it is given only to those who have worked overseas.—K. A. P.

### A Useful Disinfectant.

YES, "Rosol" is an efficient and economical disinfectant for general domestic purposes. For sinks, closets, drains, etc., from ½ oz. to 1 oz. per gallon of water is the usual strength. It may be purchased from the Rosebery Metal Works, 149-153 Rosebery Avenue, E.C. 1.—D. A.

### Decorations of Hospital-Ship Nurses.

As a Military Nursing Sister of a hospital ship carrying wounded from one of the theatres of war during the period from August 5, 1914, to December 31, 1915, you are entitled to the 1914-1915 Star. This is in accordance with a recent decision of the War Office, which also permits the awards of the British War Medal and the Victory Medal to hospital-ship sisters and nurses.—IN DOUBT.

